# Puzzle resolved: CFTR mediates chloride homeostasis by segregating absorption and secretion to different cell types

**DOI:** 10.1172/JCI174667

**Published:** 2023-10-16

**Authors:** Burkhard Tümmler

**Affiliations:** 1 Department for Pediatric Pneumology, Allergology and Neonatology, Hannover Medical School, Hannover, Germany.; 2 Biomedical Research in Endstage and Obstructive Lung Disease, German Center for Lung Research, Hannover, Germany.

## Abstract

In the lungs, the cystic fibrosis transmembrane conductance regulator (CFTR) regulates ion transport in surface-airway epithelia and submucosal glands, thus determining airway surface liquid (ASL) volume and mucus hydration. In this issue of the *JCI*, Lei Lei and colleagues report that the CFTR-rich and barttin/Cl^–^ channel–expressing ionocytes mediate chloride absorption across airway epithelia, whereas the more abundant basal cells and secretory cells mediate chloride secretion. Thus, CFTR-mediated secretion and absorption of chloride ions in the lung are segregated by cell type, which has implications for future molecular therapies for cystic fibrosis lung disease.

## CFTR and cystic fibrosis

The cystic fibrosis (CF) transmembrane conductance regulator (CFTR) is an anion channel that regulates salt and water homeostasis across epithelial membranes (1). CFTR transports chloride ions to control fluid absorption or secretion by epithelia, and moreover, it moderately conducts bicarbonate to regulate intracellular and extracellular pH. In the lungs, CFTR regulates ion transport in surface-airway epithelia and submucosal glands, thus determining airway surface liquid (ASL) volume and mucus hydration (1, 2).

Mutations in the *CFTR* gene cause CF (3), a severe generalized disease of exocrine glands characterized by mucus plugging of the exocrine ducts. Absent or abnormal CFTR predisposes to chronic airway infections, which determine morbidity and prognosis in most people with CF. Thanks to continuously improved treatment programs during the last five decades, this lethal pediatric disease has been transformed into a chronic disorder with a median life expectancy of, nowadays, more than 50 years (4).

## CFTR expression in human airways

CFTR is expressed in only a few percent of human airway epithelial cells (5, 6). In quantitative terms, secretory cells are the dominant cell type that expresses CFTR in the surface epithelium of large and small airways (5). Single-cell RNA sequencing moreover identified a population of rare cells termed ionocytes that express very high levels of CFTR (7, 8). Ionocytes constitute only about 0.3% of total cells in conducting airway epithelial cells (5, 6), but are the site of highest CFTR expression in airway cells (7, 8). The lineage of the CFTR-rich pulmonary ionocyte is specified by the transcription factor FOXI1. Besides CFTR, FOXI1 also regulates the expression of other ion transporters such as the V-ATPase proton pump (7, 8), suggesting that these cells may be key players for the water, ion, and acid-base balance of the lung.

## Role of CFTR in the pulmonary ionocyte

In this issue of the *JCI*, Lei Lei and colleagues (9) report on the role of CFTR in the ionocyte. Lei et al. transduced primary human airway epithelial cells with a lentivirus that overexpresses FOXI1 to increase ionocyte abundance. Subsequently, the cells were grown in culture using protocols for polarized epithelial cells. Lei and colleagues counted the number of ionocytes in the culture and measured the ASL volume. Compared with control cultures, which possessed 0.1% to 0.5% ionocytes, the proportion of ionocytes increased to about 4% and the ASL volume decreased. Alternatively, if the *FOXI1* gene was disrupted, the ionocytes disappeared from the culture and the ASL volume slightly increased. Thus, ionocytes mediate the absorption of ASL. However, ASL absorption was not observed if epithelial sodium channel (ENaC) activity was blocked or if *FOXI1* was transduced into airway epithelial cells from CF donors, indicating that functional ENaCs and CFTR channels are necessary to mediate liquid absorption.

**Figure 1:**
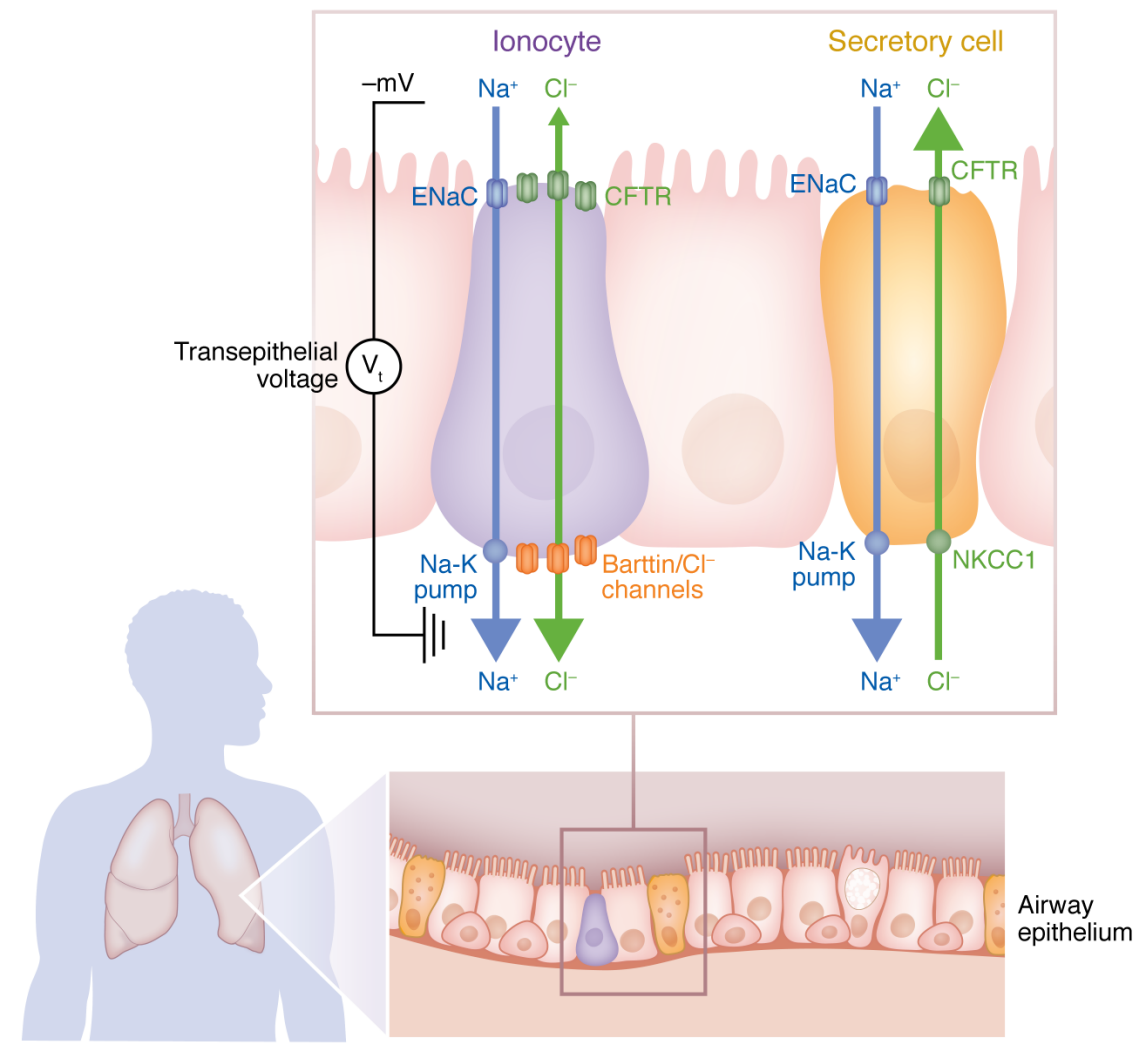
CFTR mediates chloride movement in ionocytes and secretory cells of human lungs. The airway epithelium contains few ionocytes as a percentage of total cells, but this cell type has the highest expression of CFTR in airway cells. In comparison, secretory cells are the dominant cell type that expresses CFTR in the surface epithelium. At physiological chloride concentrations, the transepithelial electrochemical gradient drives chloride absorption through the ionocyte. Apical CFTR channels and basolateral barttin/Cl^–^ channels mediate the flow of chloride ions. The electrical gradient across the airway epithelium is generated by apical ENaCs, and the basolateral Na-K pump drives chloride absorption. These characteristics allow pulmonary ionocytes to absorb liquid from the apical ASL. In contrast, secretory cells import chloride ions via basolateral NKCC1 and secrete chloride through apical CFTR channels. In these cells, apical ENaC and the Na-K pump share a pathway for absorbing sodium. Notably, CFTR serves opposite functions in different cell types. Figure adapted from Lei et al. (9).

Next, Lei and colleagues searched for the channel that mediates the transport of the chloride ions across the basolateral epithelial membrane and allows for liquid absorption. Since the protein barttin has been identified as a marker protein for ionocytes that is not present in any other airway epithelial cell (7, 8), Lei and colleagues studied the disruption or overexpression of the barttin gene *BSND* in FOXI1-overexpressing cells at the air-liquid interface. With reduced barttin levels, ASL volume increased and liquid absorption decreased, and with increased barttin levels, the opposite was observed (9). Barttin is an accessory subunit of human ClC-type chloride channels, regulating the localization, conductance, and open probability of the channel (10, 11). Lei and authors concluded that apical CFTR channels and basolateral barttin/Cl^–^ channels mediate the transcellular flow of chloride ions. At physiological chloride concentration, the electrical gradient across the airway epithelium mainly generated by apical ENaCs and the basolateral Na-K pump will drive chloride absorption through the ionocyte (Figure 1) (9). In other words, the physiological role of pulmonary ionocytes is the absorption of liquid from the apical ASL.

## Segregation of CFTR-mediated chloride secretion and absorption

Transcriptional profiling taught us that most CFTR is expressed in absolute and relative terms by secretory cells that lack barttin/Cl^–^ channels (5, 6). Thus, the secretory cells drive chloride secretion through apical CFTR channels (5) (Figure 1). In summary, the airway epithelium segregates electrolyte absorption to CFTR- and barttin/Cl^–^ channel–expressing ionocytes and electrolyte secretion to CFTR-expressing secretory and basal cells. Ion flow and the relative proportion of absorption and secretion are driven by the electrical gradient across airway epithelia and by the spatial distribution of the CFTR-expressing cell types in the lung.

The authors’ finding that CFTR-mediated secretion and absorption of chloride ions in the lung are segregated by cell type resolves an issue that gave rise to conflicting hypotheses (1, 12) about the etiology of the basic defect in CF discovered in the early 1980s (13–15) and has puzzled scientists for decades. Now we learn that opposite functions of CFTR are allocated to different cell types. Such a division of tasks may also apply to other organs affected in CF such as the sweat gland, where CFTR mediates β-adrenergic chloride secretion in the coil and chloride reabsorption in the duct, both of which are defective in CF (14, 15).

## Implications for CF

The differential roles of CFTR in ASL volume homeostasis have implications for future molecular therapies for CF lung disease. Mutations in the barttin gene *BSND* cause congenital deafness and renal failure, but are not associated with lung disease (16). Hence, the correction of CFTR-mediated absorption in ionocytes may be not necessary to cure CF lung disease. Conversely, targeting secretory and basal cells could restore CFTR-mediated secretion of chloride and bicarbonate to prevent the typical clinical symptoms of CF lung disease (5). By 2023, these options are relevant for people with CF who do not produce any mutant CFTR protein. More than 90% of people with CF, however, can nowadays benefit from the highly efficient triple therapy with elexacaftor, tezacaftor, and ivacaftor (17), which abrogates mucus plugging as the first morphological symptom of perturbed salt and water homeostasis in CF lungs (18). Systemic therapy with the CFTR modulators targets all organs affected by CF and upon drug binding, mutant CFTR adopts the conformation of wild-type CFTR protein (19). In other words, pharmacological therapy with CFTR modulators does not interfere with the delicate balance of CFTR-mediated secretion and absorption of ASL and will probably remain more efficacious in real life for the CF patient community than any of the ongoing ambitious attempts to correct the CFTR gene, transcript, or protein (20).

## Version 1 10/16/2023

Electronic publication
